# Supplementary material for: CESA: Cross-species Epitope Sequence Analysis for discovery of existing antibodies useful for phospho-specific protein detection in model species
Source: bioRxiv. 2024 Dec 22:2024.12.20.629730. Preprint. [Version 1] doi: 10.1101/2024.12.20.629730 (PMC11702626; doi:10.1101/2024.12.20.629730)

bioRxiv preprint doi: https://doi.org/10.1101/2024.12.20.629730; this version posted December 22, 2024. The copyright holder for this preprint (which was not certified by peer review) is the author/funder, who has granted bioRxiv a license to display the preprint in perpetuity. It is made available under aCC-BY 4.0 International license.

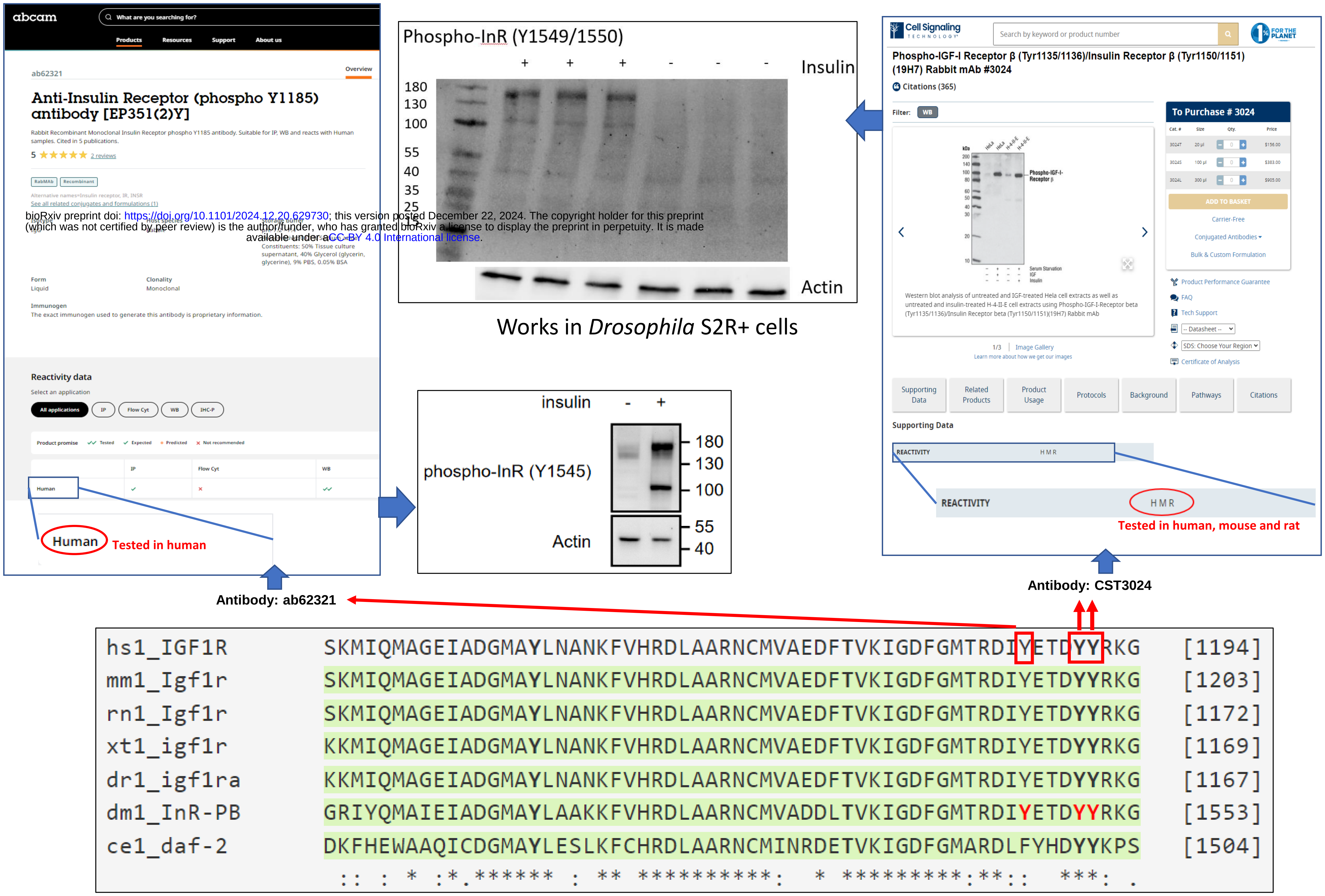

Supplement: Supplement 3 [file NIHPP2024.12.20.629730v1-supplement-3.pdf]
